# Participatory Design of an Activities-Based Collective Mentoring Program in After-School Care Settings: Connect, Promote, and Protect Program

**DOI:** 10.2196/22822

**Published:** 2021-04-12

**Authors:** Alyssa C Milton, Elizabeth Stewart, Laura Ospina-Pinillos, Tracey Davenport, Ian B Hickie

**Affiliations:** 1 Brain and Mind Centre University of Sydney Camperdown Australia; 2 Faculty of Medicine and Health University of Sydney Camperdown Australia; 3 Department of Psychiatry and Mental Health Faculty of Medicine Pontificia Universidad Javeriana Bogotá Colombia

**Keywords:** participatory design, children, after school care, health, well-being, program development, community consultation

## Abstract

**Background:**

Out of school hours care (OSHC) services provide a unique opportunity to deliver early intervention programs to enhance primary school–aged children’s social, emotional, physical, and cognitive well-being; however, such programs are currently lacking.

**Objective:**

This study aims to address the lack of well-being programs for children accessing OSHC services in the research literature by using participatory design (PD) to collaboratively develop and test an OSHC well-being program—the connect, promote, and protect program (CP3).

**Methods:**

The study employed methods of PD, user (acceptance) testing, and iterative knowledge translation to develop a novel well-being program framework—CP3—with key stakeholders (eg, children, OSHC staff, volunteers, families, clinicians, educators, and researchers). Thematic techniques were used to interpret and translate the qualitative information obtained during the research and design cycles.

**Results:**

The co-design process generated the CP3 model, which comprises a group-based mentoring approach to facilitate enhanced activities in OSHC settings. Activities are underpinned by 4 key principles of program delivery: build well-being and resilience, broaden horizons, inspire and engage, and connect communities.

**Conclusions:**

To our knowledge, the CP3 program is the first co-designed well-being program developed specifically for OSHC services. This co-design process is key to ensuring local community needs—particularly those of young people accessing OSHC—are met and that these individuals are meaningfully and actively involved in all stages of the research and design process, from conception to implementation, evaluation, and continuous improvement.

## Introduction

### Background

In the most recent report by the Australian Early Development Census (AEDC), 22% of primary school–aged children were found to be vulnerable to experiencing a developmental delay in one or more areas of functioning [1]. This included delays in social competence, emotional maturity, language and cognitive skills, communication and general knowledge, and/or physical health and well-being [1]. The rates of developmental vulnerability are reflected in other Organization for Economic Co-operation and Development (OECD) countries and have sparked international discussions on how governments, educators, individuals, and communities can work together to minimize the risk of developmental vulnerability and maximize the likelihood that all children have the best chance of a positive early start [2]. A key focus area that has arisen is the importance of using existing educational structures to optimize the environments in which children learn and grow [2]. This includes broadening the scope of educational curriculums to include programs that target children’s health and well-being and, importantly, delivering programs not only in formal school hours but also in before and after school care [3].

Out of school hours care (OSHC) services offer a safe and supervised environment for primary school–aged children before and after school. These centers provide vital services for many families by enabling parents and caregivers to achieve a balance between childcare, social responsibilities, and work [4]. In Australia, OSHCs are supported by the My Time, Our Place Framework [5], which seeks to assist services in responding to children’s needs, interests, and choices. The framework forms part of the Australian government's National Quality Framework [6], which focuses on ensuring that children receive a high standard of education and care while attending OSHC. In addition, OSHC offers a unique opportunity to implement extracurricular programs designed to enhance children’s health and well-being in a multidimensional way, including socially, emotionally, physically, and cognitively [7]. However, despite their potential, OSHCs often function as supervised childcare facilities, resulting in a missed opportunity to implement prevention and early intervention programs [8]. As such, there has been increased attention from researchers, educators, the government, and the broader community into how specific well-being–focused programs delivered during out of school hours could be better used to support children’s learning and growth.

Globally, there is currently a dearth of literature on how health and well-being programs for primary school–aged children can be developed, implemented, and evaluated in OSHC settings. Although numerous programs have been developed to target adolescent groups [9], far less research has been conducted examining health and well-being programs to support children in the primary school years (aged 5 -11 years), aptly named the *in-betweeners*, as they fall in between the toddler and postpubertal groups [10]. Programs developed for these *in-betweeners* have been overwhelmingly skewed toward physical health and nutrition [11,12], and although interventions targeting healthy eating and physical activity are undoubtedly beneficial, they fail to consider children’s health more holistically. Moreover, many existing programs have tended to be highly specific and nongeneralizable, providing limited scope beyond the implementation of the program itself [13,14]. Such programs at this age are critical, as experiences from early to middle childhood, including a child’s environment and relationships, shape their brain development and lay the foundations for their future social, emotional, cognitive, and physical well-being [15-17]. Disruptions in this developmental process can have long-term impacts, affecting the way children learn and interact with others [18].

In OSHC services, the provision of high-quality programming, characterized by positive staff-child relationships, a variety of enrichment activities, and children’s choice and input into program activities, has been positively associated with children’s engagement and motivation [19-21] as well as their cognitive and social outcomes [22]. The presence of appropriately trained staff and out-of-school coordinators to assist with professional development and networking are other factors related to OSHC quality [23]. Given that OSHC services differ in geographic location, expertise of staff, and the characteristics and number of children who attend, programs that are suitable for one OSHC service may not be feasible or appropriate for another. As such, providing a model that allows OSHC programs to be individually tailored to meet the needs and preferences of children and their families, the skill set of staff, and broader ethos and goals of the community is critical.

At present, there are no clear models in the literature detailing how well-being–focused programs, including appropriate mentorship and program development, can be developed and delivered in OSHC settings. As such, there is an urgent need to develop an evidence-based framework to guide staff, educators, community members, and other key stakeholders who are responsible for the delivery of well-being–focused programs to children in primary school years. To develop a program framework that best meets the needs of the community and service, the involvement of key stakeholders (eg, children, parents and caregivers, staff, volunteers, educators, clinicians, and community members) in the co-design and evaluation of the intervention is critical [7].

One way to develop this model is through the use of participatory design (PD) research methods, also known as co-design, in which stakeholders are placed at the center of the design process [24,25]. Often used in designing digital technologies, PD is part of a paradigm shift toward collaborative bottom-up engagement, whereby stakeholders jointly explore and create solutions to program design and service delivery. The PD process involves a series of iterative design cycles in which all stakeholders contribute their knowledge to produce a program model [25,26]. The ideas generated within each cycle are discussed, evaluated, and built upon during the subsequent design phases. Importantly, all stakeholders participate in each development cycle [24], as they share equal responsibility with the research team for outcomes [27]. This iterative research design cycle of development, feasibility, evaluation, and implementation follows the Medical Research Council guidelines for developing complex interventions [28].

### Objectives

The primary aim of this study is to use a multidisciplinary collaboration between members of an OSHC community (eg, staff, volunteers, parents, and caregivers), local community members (eg, youth workers from local organizations, clinicians, and educators), and researchers to co-design a well-being program model for delivery in OSHC settings. The program has been termed the connect, promote, and protect program (CP3).

## Methods

### Ethics

This research was approved by the University of Sydney’s Human Research Ethics Committee (protocol numbers: 2017/509 AND 2018/832).

### Study Design

**Figure 1:**
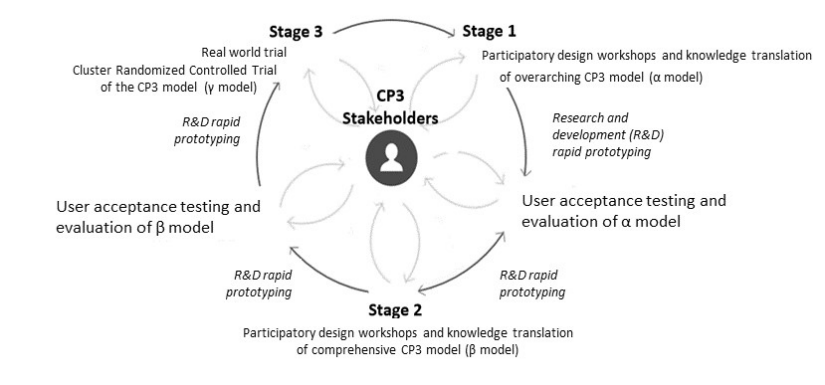
Connect, promote, and protect program research and development cycle. CP3: connect, promote, and protect program; R&D: research and development.

This study employed a prospective observational design, including PD and user (acceptance) testing methodologies. The research and development cycle was conducted in a series of stages based on previously established processes in the academic literature [25,29]. The co-design and build of CP3 included several iterative stages that were built upon each other (Figure 1). This research reports on stage 1, which involved PD workshops and knowledge translation, whereby knowledge and ideas generated during workshops were translated to produce an overarching CP3 program model (α model). Stages 2 and 3 and in train will be reported elsewhere in the future.

### Participants

Adult participants were recruited from a community sample in Illawarra, New South Wales region, between July 2017 and September 2018. Electronic and paper-based advertising materials were used to notify potential participants of the study. Passive snowballing through the networks of identified participants was also used to increase the participant pool [30]. Participants comprised 3 main stakeholder groups: (1) parents, guardians, or primary carers of primary school children; (2) volunteers or employees of the nongovernment organization establishing the OSHC; and (3) stakeholders such as local community members, supportive others (such as grandparents, aunties, or uncles), academics, educators, and school personnel from Illawarra (where the program was to be established). The inclusion criteria were as follows: (1) identification as part of one of the main stakeholder groups; (2) ability to participate in English; and (3) provision of written informed consent to participate in the research. Participants did not receive any compensation or reward for participating in the workshops; however, all workshops were catered.

### PD Workshops

A total of four 3-hour PD workshops were held at the OSHC, where the program was initially piloted. The PD workshops were facilitated by a psychologist (AM) and co-facilitated by a second researcher. Co-facilitators had experience in either the OSHC sector or youth mental health (LOP, SP, RA, and NA). A scribe was present in each PD workshop to take detailed notes. Within each PD workshop, adult stakeholder backgrounds were intentionally mixed, meaning that parents and guardians, volunteers or employees, and other community stakeholders all participated together. This mixed participant approach enriches the workshop discussion by drawing on a range of participant experiences, ultimately enhancing the overall program design solution [31].

In line with other academic literature, the workshop agenda includes 3 phases: discovery, evaluation, and prototyping [25,31,32]. In the discovery phase, stakeholders were involved in the design process by identifying local needs and issues and defining research objectives, strategies, and goals. These discussions help to identify key issues and shape creative concepts and ideas for program development and implementation. In the evaluation phase, stakeholders worked together to evaluate program ideas (whether they are ideas from external sources such as other programs or those generated in previous workshops) to understand how they might be improved and refined to fit the local program needs. In the prototyping phase, stakeholders collaborated to develop and refine content and work through implementation strategies to determine the optimal program design.

Workshop sessions applied an iterative knowledge translation process so that preliminary ideas generated within earlier workshops were further developed (and fed back on) by participants in later workshops.

### Data Analysis

Qualitative data sources (artifacts) from PD workshops included detailed notes from the scribe and notes written by participants on handouts, worksheets, and surveys. All data were uploaded to the NVivo (QSR international, version 11) software. Qualitative data were interpreted using previously established thematic techniques [33] by 2 researchers (AM and NA). All qualitative data sources from the workshops and feedback surveys were reviewed by noting the relevant points. Key concepts were subsequently analyzed across all participants to develop an initial coding framework. Notes were then coded in NVivo [34] using this framework by 2 researchers per transcript. The coding followed an iterative process of reading, coding, and discussing the pattern and content of the coded data. Similarities and differences in opinion were discussed until a consensus was reached. An initial report was written for the knowledge translation team, who then established the CP3 model for user acceptance testing and evaluation. The knowledge translation process involves researchers working with stakeholders to synthesize, exchange, and apply knowledge to enhance systems and improve outcomes [35].

### Compliance With Ethical Standards

All procedures performed in studies involving human participants were in accordance with the ethical standards of the institutional and/or national research committee (including the name of committee+reference number) and with the 1964 Helsinki declaration and its later amendments or comparable ethical standards.

### Informed Consent

All individuals completed an informed consent form before participating in the study. All data, including images and figures in this publication, are presented in nonidentifiable formats.

## Results

### Sample Characteristics

In total, 28 participants took part in the initial 3 workshops during August and September 2017, and a further 6 adult participants took part in 2018. The demographic characteristics of participants are presented in Table 1 (see Multimedia Appendix 1 for a full breakdown of participant characteristics for individual workshops).

**Table 1:** Basic participant demographics.

Demographic item			Values
Population, N			34
**Detailed participant type^a^, n (%)**			
	Parent, guardian, or primary carer of a primary school–aged child	8 (24)	
	Community volunteers	4 (12)	
	Supportive other of a primary school–aged child	1 (3)	
	Potential future mentor of CP3^b^	8 (24)	
	Researcher or academic	1 (3)	
	Teacher or educator	10 (29)	
	Local community member	19 (56)	
	Other child-focused community organization	9 (26)	
**Age range (years), n (%)**			
	16-24	3 (9)	
	25-34	2 (6)	
	35-44	6 (18)	
	45-54	6 (18)	
	55-64	6 (18)	
	≥65	4 (12)	
	Did not answer	7 (21)	
**Gender, n (%)**			
	Male	11 (32)	
	Female	23 (68)	
**Language spoken at home^a^, n (%)**			
	English	27 (79)	
	Other	4 (12)	
	Did not answer	6 (18)	

^a^Multiple response options provided.

^b^CP3: connect, promote, and protect program.

### CP3 Principles

#### Discovery of CP3 Principles

In the discovery phase, which focused on creating CP3 principles, stakeholders chiefly identified the program goals. A total of 4 key themes were generated, which related to (1) enhancing well-being (*build well-being and resilience*), (2) creating opportunities for development and growth (*broaden horizons*), (3) meaningfully engaging children (*inspire and engage*), and (4) promoting social and community connectedness (*connect communities*).

**Figure 2:**
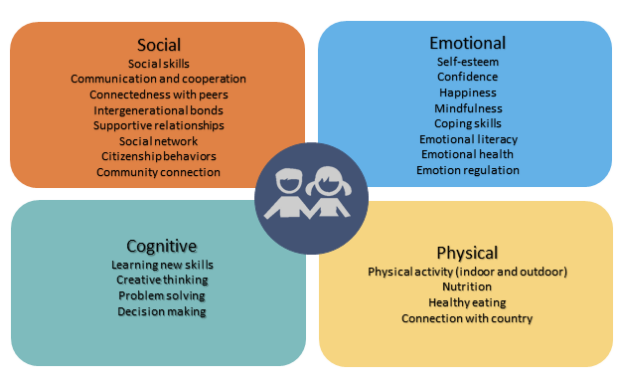
Children’s wellbeing domains.

Workshop participants emphasized that CP3 should aim to enhance children’s well-being in a multidimensional and holistic way. The multiple ideas generated relating to improving well-being were categorized into 4 key domains: social, emotional, physical, and cognitive well-being (Figure 2). Enhancing the child’s social well-being was the most frequently referenced domain, followed by emotional well-being, cognitive well-being, and physical well-being. Social well-being items included building communication and social skills, enhancing citizenship behaviors, promoting positive and supportive relationships, and feeling connected to the local community. The focus of emotional well-being is related to building self-esteem, confidence, happiness, emotional health, resilience, and coping skills. Cognitive well-being items are chiefly related to problem solving and decision making. Physical well-being items predominately focused on healthy eating, undertaking physical activity (indoor and outdoor), connecting with the environment, and understanding the benefits of healthy lifestyles.

The theme relating to broadening the child’s opportunities and skills by providing a diverse range of experiences that children might not generally have access to in their day-to-day lives was highlighted in all workshops. Participants emphasized that the activities on offer in CP3 should be enriching in that they help primary school–aged children broaden their horizons, develop new skills, and contribute to their personal and social development.

The theme related to meaningfully engaging children had a number of different areas of focus. Consistent themes raised in the workshops related to the best approach to facilitating CP3 chiefly centered around flexibility and choice for children; “...giving the children some freedom to choose what activities they enjoy” (OSHC manager, workshop 2) was viewed as important as it was reported to be “...nearly impossible to expect all children to engage in a controlled activity after a long day at school, especially if they are not interested in it” (OSHC manager, workshop 2*)*. This flexibility included the children helping to provide input and co-design into what the activities program would look like: “It would be great if the activities could be tailored to the child as much as possible and be child-led. Child input and choice is important as is flexibility in programming” (community member, workshop 3).

Although the importance of social connection was also raised as part of the well-being component, participants in all workshops emphasized that enhancing social connectedness would be an important focus for CP3 as a distinct principle—not only for children accessing CP3 but also for families connected to CP3, staff and volunteers delivering CP3, and the wider community. It was hypothesized that if the program could build social connectedness, it would also create more awareness, tolerance, and understanding in the local communities through contact with others. The program would need to establish firm pathways to community resources (including people, organizations, and web-based resources) for children, their families, and the staff and volunteers delivering CP3. These community resources could range, for example, from skill development to mental health resources and services (such as counseling).

#### Prototyping the CP3 Principles

The prototyping phase led to the full formation of 4 key CP3 principles and the definitions (presented in Textbox 1), which are underpinned by the existing *My Time Our Place Framework* [5] and the *National Quality Standards* [6].

**Connect, promote, and protect program principles.**

Build well-being and resilience

- Provide activities that seek to promote and enhance children’s social, emotional, cognitive, and physical well-being

Broaden horizons

- Broaden opportunities and skills by providing a diverse range of experiences that children might not generally have access to in their day-to-day lives

Inspire and engage

- Focus on creating a spark in children as the activity is interesting, motivating, and fosters a growth mindset. Encourage meaningful involvement by promoting children’s leadership, decision making, and choice

Connect communities

- Promote connectedness, communication, and belonging as children—and their families—forge strong links with local resources and their community

### CP3 Core Program Features

#### Discovery

In the discovery phase relating to program design, stakeholders chiefly identified 2 key features of CP3: (1) group-based (collective) mentoring and (2) the provision of enhanced activities.

#### Evaluation

In the iterative evaluation phase, the provision of a mentoring component forming part of CP3 was viewed as highly acceptable across all workshops. A number of participants also highlighted that the key differentiation between CP3 and regular OSHC programming would be this mentoring component, which would require considerable focus to establish and sustain in the future:

The real point of difference of the program is the mentoring component, [we] need to capitalize on this and ensure that the program doesn’t just turn into another OSHC.

Community worker, workshop 3

The value of mentoring was also highlighted throughout the workshops:

Including the mentoring component in the program might have positive impacts for the wider community, as it plants the seed for growth and can broaden perspectives.

Community member, workshop 2

The mentoring component was not only seen as beneficial to the children accessing the OSHC but also viewed as giving the mentors themselves skills, confidence, social connection, and “a feeling of ‘giving back’” (mentoring benefits artifact, workshop 3).

Concerns were raised about child protection, and an emphasis was placed on the need to ensure that the program uses “...the right people in the right capacity” (mentoring mind map artifact, workshop 3). It was the prevailing view that such issues could be addressed through rigorous mentor recruitment, training, supervision, policies, and procedures.

In all workshops, the suggestions generated by participants highlighted that the OSHC activities on offer in CP3 should be enriched and enhanced, especially when compared with regular OSHC services. The term created for this component by participants in early workshops was *enhanced activities* as they are “...more than just extracurricular activities” (parent and community worker, workshop 1), which was subsequently accepted and adopted in the later workshops. Enhanced activities were viewed as the vehicle for carrying out the CP3 principle of *broaden horizons*—as the activities would be enriched, allow children to develop new skills, and contribute to their personal and social development. Some participants viewed this program component as particularly beneficial for more vulnerable children who might access CP3:

Enhanced activities would be wonderful. Especially as they can be completely out of reach for some young people.

Parent and community worker, workshop 1

Enhanced activities were viewed as needing to be stimulating to ensure that the children were engaged and motivated to take part. This was directly related to the CP3 principle of *inspire and engage* and went hand-in-hand with the mentoring component: “The mentoring and activities should create a spark for the child” (school teacher, workshop 3).

The overarching, iterative feedback generated during the workshops was chiefly positive:

This type of program could have huge benefits for wider community change as it sets out to make positive community connections—this can be powerful on a large scale and be a catalyst for huge community change.

Community worker, workshop 3

#### Prototyping

When prototyping the mentoring component design, participants developed a plan for group-based (collective) mentoring, otherwise defined as collective mentoring. The collective mentoring of children in group settings was viewed as more beneficial in an OSHC environment, compared with one-on-one mentoring, as it addressed concerns relating to program acceptability, matching children with mentors, mentor recruitment, and turnover, and this could easily run alongside general OSHC activities.

To enhance mentoring options for the children accessing OSHC and ensure CP3 was not a “...blanket one size fits all program...” (school teacher, workshop 3), a 3-level approach to mentoring was generated during workshop discussions. This included skill-based mentoring, CP3 mentoring, and peer-to-peer mentoring. Skill-based mentoring meant that mentors with special skills would facilitate activities in their area of expertise. It was highlighted that these “*...*mentors should be passionate about what they are teaching*...*” (school teacher, workshop 3) to motivate, inspire, and engage children in CP3*.* The second type of mentor identified was a *CP3 mentor*, trained in CP3 principles, and could provide support to the enhanced group-based activities as well as the OSHC’s day-to-day running. Peer-to-peer mentoring was also proposed as an additional avenue for CP3 to engage primary school children attending OSHC to take on a leadership role, which reflected the *inspire and engage* CP3 principle.

Specialized CP3 training, designed for both staff and volunteer mentors, was seen as crucial to the delivery of CP3. Prototyped areas of training included vision and mission of CP3; mentoring processes and relationships; building emotional literacy; child development; working with special needs; managing challenging behaviors and situations; referral pathways and support; and risk management and safety.

When prototyping the enhanced activity component, participants highlighted that during the implementation of CP3, the program would need to avoid activities being delivered in a “piecemeal manner...” (teacher, workshop 1), that is, there needed to be a coherent structure to the program, where activities link together to form a greater purpose of working toward the CP3 principles:

The building blocks system or foundation as part of the program—where it’s not just one lesson and then move on will be important. It needs a framework that everyone is privy to.

Educator, workshop 1

**Figure 3:**
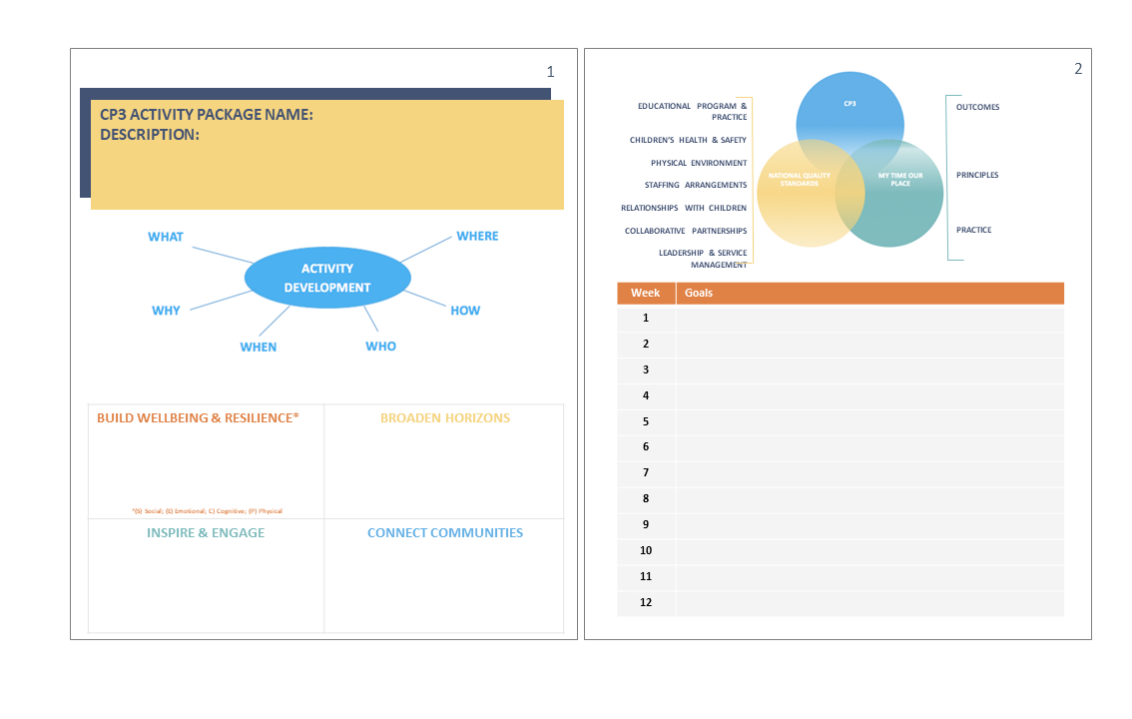
Example page from the connect, promote, and protect program activity development guide after prototyping and knowledge translation.

On the basis of this feedback, a CP3 activity development guide was prototyped. This is a tool for selecting and designing enhanced activities. It ensures that the staff and children think purposefully about programming so that it provides every opportunity to enhance the experience in terms of the CP3 principles, the *My Time Our Place Framework* and the *National Quality Standards*. The tool also supports reflective practice and sharing of ideas. An example summary page from the CP3 activity development guide is provided in Figure 3.

### Additional Program Features

#### Discovery

A total of 2 additional features of CP3 were identified, which included the provision of one-on-one well-being support for children with greater needs and involving families meaningfully.

#### Evaluation and Prototyping

The idea generated by participants that CP3 could provide additional one-on-one psychological support for children with additional biopsychosocial needs, such as “...if there was a grief issue or if there was a diagnosis that required further support...” (teacher, workshop 1), received positive feedback when iteratively evaluated. Participants emphasized that if additional support was offered, it would need to be carried out by a registered psychologist or other qualified health professionals. The provision of such additional support was seen as particularly beneficial for the prevention and early intervention of social, emotional, physical, or cognitive difficulties.

Participants also recommended that “...there needs to be a whole family approach...” (workshop 3, community member) for CP3 implementation. Ideas generated included CP3 “*...*build[ing] the capacity of parents*...*” (parent and community worker, workshop 1), which included developing a resource kit for parents, providing support pathways and “*...*link[ing] parents with counseling services*...*” (community worker, workshop 2), “*...*resources to support their children effectively*...*” (teacher, workshop 3), such as “*...*active parenting programs*...*” (teacher, workshop 3), “*...*positive parenting programs or circles of security*...*” (parent, workshop 2)*.* Providing clear communication channels such as a “*...*feedback cycle between the child, families and school*...”* (CP3 mindmap artifact, workshop 3), finding out “*...*positives about their children through feedback from the program*...*” (parent program outcomes artifact, workshop 2), telling parents “... about the focus of the learnings*...* for example, we are going to talk about character and strength this week*...*” (community member, workshop 3), and creating a CP3 newsletter or social media page (eg, Facebook) was recommended. Third, building a sense of community for parents, such as providing a “*...*chance to meet and interact with others of similar interests, problems etc...” (parent program outcomes artifact, workshop 2) and having an “*...*open day*...*” (community worker, workshop 3).

### Knowledge Translation

**Figure 4:**
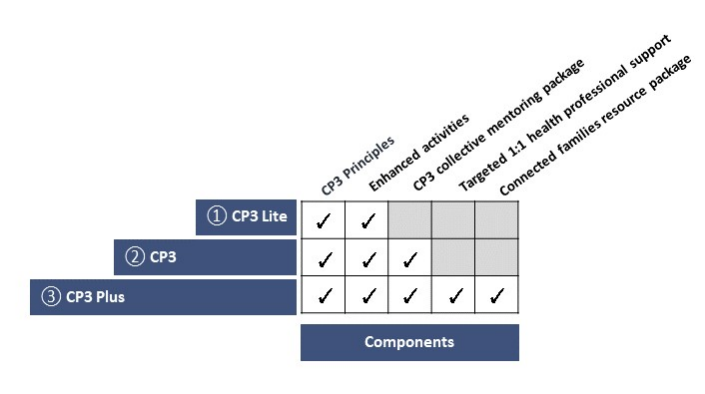
Components of connect, promote, and protect program stages. CP3: connect, promote, and protect program.

A stepped approach to implementation was raised as a possibility in the workshops for the development and evaluation of CP3. In the knowledge translation phase, this idea was refined into 3 components: CP3 Lite, CP3, and CP3 Plus (outlined in Figure 4). These components can be implemented in a stepwise manner and are now being iteratively developed, delivered, and evaluated through a formative evaluation implementation process.

**Figure 5:**
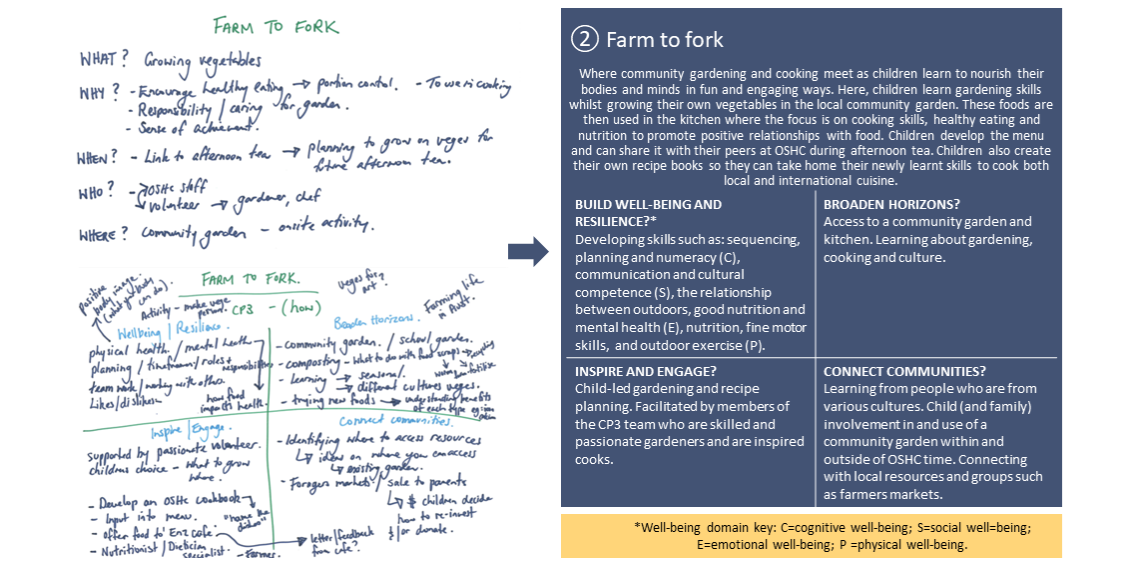
Example excerpts from connect, promote, and protect program enhanced activity planning.

CP3 Lite is the minimal viable product of CP3 (α-build). This component is the first implementation step and provides enhanced activities underpinned by the CP3 principles (*build well-being and resilience,* *broaden horizons*, *inspire and engage*, and *connect communities*) using the CP3 activity development guide. CP3 Lite is facilitated by OSHC educators and qualified community experts. Example excerpts from the CP3 activity planning process, which led to the establishment of the CP3 activity development guide for training and trialing, are presented in Figure 5.

**Figure 6:**
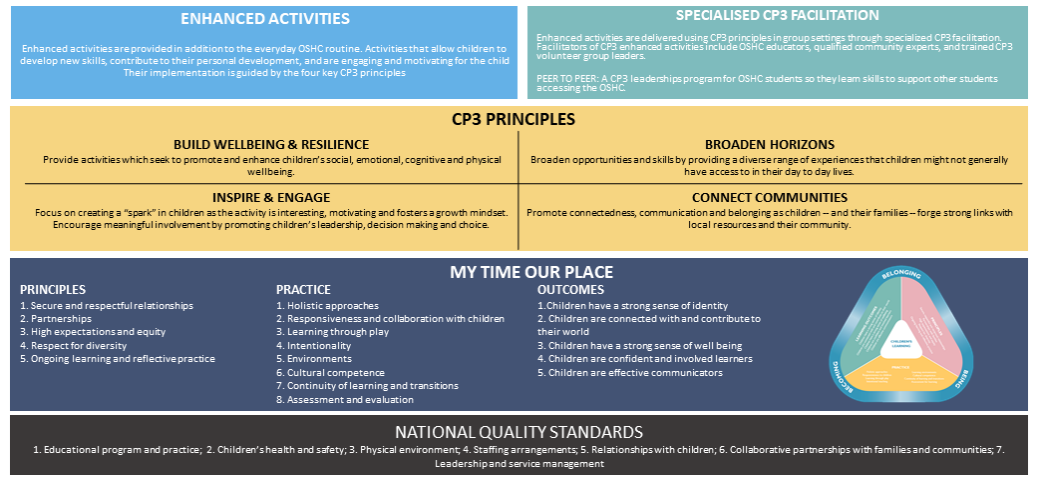
Connect, promote, and protect program model underpinned by the existing My Time Our Place Framework and the National Quality Standards. CP3: connect, promote, and protect program.

The next component is the implementation of CP3 (Figure 6), which is underpinned by the existing *My Time Our Place Framework* [5] and the *National Quality Standards* [6] that are used in OSHC services. This includes the facilitation of enhanced activities and a fully developed collective mentoring component. This component includes the development of a training package for CP3 volunteers to aid staff in facilitating CP3 and may also use peer-to-peer support. The final component, CP3 Plus, is implemented as the final step and provides enhanced activities, collective mentoring and the additional family resource package, and one-on-one support. Ultimately, service evaluation outcomes determine the need, utilization, and effectiveness of these components.

## Discussion

### Principal Findings

In this study, we used PD (or co-design) research methods to develop a novel health and well-being program for primary school–aged children (aged 5-12 years) to be delivered in OSHC: CP3. To our knowledge, CP3 is the first health and well-being program model designed specifically for OSHC settings that allows tailored interventions to be developed depending on the unique needs and preferences of the end users, including children (in later stages), their parents and guardians, staff, volunteers, and the broader community. CP3 adopts a holistic, community-focused approach, encouraging active participation of community members, peer-to-peer and adult-led mentoring, and interventions that not only focus on physical development but also foster social, emotional, and cognitive well-being. In this way, CP3 addresses the goals and objectives of the AEDC [36] and OECD [2] for early childhood education and care, which focus on building supportive environments and developing strength-based programs to build children’s competencies during primary school years.

CP3 addresses a major gap in the literature and in the delivery of universal health and well-being programs in educational settings. Unlike existing OSHC programs, which tend to be prescriptive, narrowly focused, and nongeneralizable, CP3 offers a framework for flexible program development and delivery while ensuring that a high standard of program development will be maintained. The 4 CP3 principles co-designed during PD workshops (ie, *build well-being and resilience, broaden horizons, inspire and engage, and connect communities*) ensure that the goals of CP3 interventions can be clearly delineated. This is critical, as one of the pitfalls in the implementation of new well-being programs is that they often fail to adhere to the core components of best practice and frequently do not use a program model [37,38]. Moreover, as highlighted in the Medical Research Council guidelines for developing complex interventions, the first step to developing novel interventions is the identification or development of a theoretical model, which this study has achieved [28]. In addition, CP3 provides more specific guidance on essential program features, namely collective mentoring and enhanced activities. The involvement of mentors is a key point of difference between CP3 and existing OSHC programs and promotes the CP3 principle of *connect communities.* Currently, the available evidence in the literature indicates that for a program to be effective, it is necessary to follow best practices in recruiting, training, and providing ongoing support and supervision to mentors [37,39]. The views were generated by participants in the PD workshops, particularly because of the importance of child protection when delivering the program. Such support for mentors may also assist them in building and sustaining their relationship with the OSHC over an extended period, as high staff turnover can negatively impact engagement [40].

CP3 has been designed to ensure universal access to a health- and well-being–focused program for all children, meaning equal opportunities and adequate fit regardless of socioeconomic background, geographic location, community resources, goals and expertise of service providers, and preferences and needs of the community. Therefore, one of the major advantages of CP3 is its appropriateness and ability to adapt to disadvantaged and vulnerable groups, such as children from low socioeconomic backgrounds, geographically isolated communities, Aboriginal and/or Torres Strait Islander people, and people from culturally and linguistically diverse groups. By placing communities at the center of the design and development process, CP3 ensures that interventions will be culturally sensitive and relevant, will respect local knowledge and meaning, and will empower communities to take action by taking matters into their own hands. This community-based approach transitions power back to local communities and is central to allowing communities and, subsequently, their young people to thrive.

Despite the goal of universal access and participation, research has shown that the simple introduction of a universal program does not in itself guarantee equal access or equal participation [41]. Therefore, one of the mandates of the CP3 coordinator role is to assist families and communities with greater socioeconomic challenges to actively participate in both the design of the program and using OSHC services. This is important as research and evaluations of OSHC programs have found greater positive effects on outcomes for at-risk populations compared with more heterogeneous samples [42,43]. The success of the universal program approach to design and delivery will be further evaluated during the full program evaluation, which will take into account both service-specific and external factors such as the Australian government changes to parent activity testing and childcare subsidies introduced in 2018 [44].

### Strengths and Limitations of the Research

A current limitation is that this study reports on the development of the CP3 program only. Future research is required to ensure a robust evidence base. Stage 2 of the project is currently being conducted (July 2020 to June 2021), which involves iterative user (acceptance) testing via a naturalistic formative service evaluation of the implementation CP3 combined with further PD workshops. This stage will test and refine the ideas generated in stage 1 in partnership with a wider group of stakeholders associated with the OSHC (ie, also include the children attending the OSHC) to inform a more comprehensive CP3 model (β model). In the future, stage 3, a real-world cluster randomized controlled trial will be carried out on the CP3 model (β model).

In designing the CP3 α model, an iterative PD approach was employed that placed key stakeholders at the center of the design and development process. This process of co-design and development will continue to be used, as CP3 is implemented and evaluated in stages 2 and 3. These co-design research methodologies are also embedded in the program design itself in the continuous process of re-evaluation and re-responding to community needs as children and their communities grow and change over time. For instance, the CP3 principles of community collaboration (*connect communities*) and meaningfully engaging children in the decision-making process (*inspire and engage*) emphasize the importance of engaging end users at all stages of the intervention development process. Children themselves form part of the co-design process; however, this research is still underway, as it forms part of the evaluation and thus will be reported elsewhere. This co‐design and collaborative management means that the OSHC can be delivered according to the communities’ strengths while ensuring that the level of program consistency is maintained. Despite these benefits, the use of PD methods is also challenging. For example, in this research, PD workshops could only take place in English because of budget limitations, that is, this research did not have funds to provide translators and to translate all study materials (such as consent forms and participant information statements). This may limit the generalizability of the research, although people who spoke English as a second language participated. Interestingly, the percentage of individuals who only speak English at home (7/34, 79%) accurately reflected the demographics of the Illawarra region (80.6%) [45]. Furthermore, the PD process takes considerable time and commitment from OSHC staff, researchers, and the wider community. Academics designing a well-being program to be delivered and evaluated without input from a wider group of stakeholders would certainly be less time intensive; however, this would take away from the deep understanding and ability to respond to local community needs, which arguably leads to a better program.

Research suggests that health programs can take up to 17 years to move 14% of original research into actual service delivery [46]. However, here the use of an ongoing formative evaluation process allows for the program design to be agile and actively respond to local needs as they arise over time. For example, when new opportunities arise (such as when mentors or staff with particular skills are recruited), additional enhanced activities can be designed using the CP3 activity development guide, which is guided by CP3 principles, the *My Time Our Place Framework* [5] and the *National Quality Standards* [6]. Using this approach, the CP3 model can grow and be improved in real time. This iterative design cycle of development, feasibility, evaluation, and implementation follows recommendations by the Medical Research Council’s newer guidelines for developing complex interventions [28].

### Formative and Future Evaluation of CP3

CP3 is currently undergoing a formative evaluation, and plans are being made for future full-scale evaluation. These evaluation stages of research are crucial, as research suggests that many new mentoring programs are pursued without any supporting evidence from reliable or valid process or outcome evaluations [37,38]. Furthermore, research into what collective (group based) mentoring with enhanced activities has not, to our knowledge, been investigated either within or outside of OSHC settings. Therefore, future evaluation of outcomes will influence the proliferation of this type of program. Finally, one-on-one mentoring interventions that use evidence-based practices and provide the child with long-term, high-quality relationships (as a stand-alone one-on-one mentoring intervention or in combination with structured activities) can yield small but positive improvements in a range of psychosocial, health behavior, and academic outcomes [37,38,47]. However, lower quality one-on-one mentoring interventions can negatively impact children. Thus, ensuring that CP3 applies high-quality programming and has an evidence base is vital.

Additional PD with children at multiple OSHC sites will occur from 2019 to 2021 as part of the formative evaluation of CP3 and thus are yet to be reported. Further plans are also being made to measure the effectiveness of the CP3 model in a large-scale randomized controlled cluster trial. The major challenge is ensuring that engagement continues to be high when research extends to new sites. There is a possibility that successful PD engagement is because of the nuances of the pilot OSHC community. For example, the first pilot OSHC site for CP3 was a brand new service; thus, a focus on culture change to move away from a traditional OSHC model toward the CP3 is not required, whereas other already-established OSHC *early adopter* sites may require a different focus. Specifically, the need for effective staff by in and change management may be required when CP3 is introduced into already-operational OSHC sites. Ultimately, the competence and capacity of local facilitators will be crucial for successful implementation. This will be evaluated as CP3 is rolled out further in already-established OSHC sites.

### Conclusions

To our knowledge, CP3 is the first co-designed health and well-being program to be delivered to primary school–aged children in an OSHC setting. The co-design process is key to ensuring that local community needs are met and that they are meaningfully and actively involved in all stages of the research and design process, from conception to implementation, evaluation, and continuous improvement. By providing a framework that encourages tailored interventions to be developed depending on the unique needs and preferences of the end users (eg, children and their families, staff, volunteers, and the broader community), CP3 takes an important step forward toward achieving universal access to a holistic health and well-being program for all children. The CP3 model is currently under evaluation, and the results will be used to determine the overall success and inform ongoing development and implementation.

## Appendix

Multimedia Appendix 1 Basic participant demographics.

## Abbreviations

AEDC: Australian Early Development Census
BMC: Brain and Mind Centre
CP3: connect, promote, and protect program
OECD: Organization for Economic Co-operation and Development
OSHC: out of school hours care
PD: participatory design
