# Supplementary material for: Participatory Design of an Activities-Based Collective Mentoring Program in After-School Care Settings: Connect, Promote, and Protect Program
Source: JMIR Pediatr Parent. 2021 Apr 12;4(2):e22822. doi: 10.2196/22822 (PMC8076982; doi:10.2196/22822)
Supplement: Multimedia Appendix 1 [file pediatrics_v4i2e22822_app1.docx]

**Supplementary Files**

**Table 1.** Basic participant demographics.

| **Workshop** | **1** | **2** | **3** | **4** | **Total** |
| --- | --- | --- | --- | --- | --- |
|  | **n(%)** | **n(%)** | **n(%)** | **n(%)** | **n(%)** |
| **Total N** | **14** | **6** | **8** | **6** | **34** |
| Participant type^a^ |  |  |  |  |  |
| *Local community member* | 9 (82%) | 4 (80%) | 6 (86%) | 0 (0%) | 19 (56%) |
| *Parent/ guardian/ primary carer of a primary school student* | 3 (27%) | 1 (20%) | 2 (29%) | 2 (33%) | 8 (24%) |
| *Potential future mentor of CP3* | 1 (9%) | 2 (40%) | 1 (14%) | 4 (66%) | 8 (24%) |
| *Researcher / academic* | 1 (9%) | 0 (0%) | 0 (0%) | 0 (0%) | 1 (3%) |
| *Supportive other of a primary school student* | 0 (0%) | 0 (0%) | 1 (14%) | 0 (0%) | 1 (3%) |
| *Teacher/ educator* | 2 (18%) | 3 (60%) | 1 (14%) | 4 (66%) | 10 (29%) |
| *Community volunteer* | 3 (27%) | 0 (0%) | 1 (14%) | 0 (0%) | 4 (12%) |
| *Other child community organisation* | 4 (36%) | 0 (0%) | 3 (43%) | 2 (33%) | 9 (26%) |
|  |  |  |  |  |  |
| Age range |  |  |  |  |  |
| *16-24 years* | 1 (7%) | 0 (0%) | 0 (0%) | 2 (33%) | 3 (9%) |
| *25-34 years* | 0 (0%) | 0 (0%) | 1 (13%) | 1 (17%) | 2 (6%) |
| *35-44 years* | 1 (7%) | 2 (33%) | 1 (13%) | 2 (33%) | 6 (18%) |
| *45-54 years* | 2 (14%) | 1 (17%) | 2 (25%) | 1 (17%) | 6 (18%) |
| *55-64 years* | 3 (21%) | 2 (33%) | 1 (13%) | 0 (0%) | 6 (18%) |
| *65 years plus* | 1 (7%) | 0 (0%) | 3 (38%) | 0 (0%) | 4 (12%) |
| *Did not answer* | 6 (43%) | 1 (17%) | 0 (0%) | 0 (0%) | 7 (21%) |
|  |  |  |  |  |  |
| Gender |  |  |  |  |  |
| *Male* | 5 (36%) | 3 (50%) | 3 (38%) | 0 (0%) | 11 (32%) |
| *Female* | 9 (64%) | 3 (50%) | 5 (62%) | 6 (100%) | 23 (68%) |
|  |  |  |  |  |  |
| Language spoken at home |  |  |  |  |  |
| *English* | 8 (57%) | 5 (100%) | 8 (100%) | 6 (100%) | 27 (79%) |
| *Other* | 0 (0%) | 1 (17%) | 0 (0%) | 3 (50%) | 4 (12%) |
| *Did not answer* | 6 (43%) | 0 (0%) | 0 (0%) | 0 (0%) | 6 (18%) |

Multiple response options provided
